# Autophagy Receptor p62 Regulates SARS-CoV-2-Induced Inflammation in COVID-19

**DOI:** 10.3390/cells12091282

**Published:** 2023-04-28

**Authors:** Verica Paunovic, Ljubica Vucicevic, Maja Misirkic Marjanovic, Vladimir Perovic, Biljana Ristic, Mihajlo Bosnjak, Milos Mandic, Danijela Stevanovic, Ljubica Harhaji-Trajkovic, Jovan Lalosevic, Milos Nikolic, Branka Bonaci-Nikolic, Vladimir Trajkovic

**Affiliations:** 1 Institute of Microbiology and Immunology, Faculty of Medicine, University of Belgrade, Dr. Subotica 1, 11000 Belgrade, Serbia; 2 Department of Neurophysiology, Institute for Biological Research “Sinisa Stankovic”—National Institute of Republic of Serbia, University of Belgrade, Bulevar despota Stefana 142, 11000 Belgrade, Serbia; 3 Clinic of Dermatovenereology, University Clinical Center of Serbia, Pasterova 2, 11000 Belgrade, Serbia; 4 Faculty of Medicine, University of Belgrade, Dr. Subotica 8, 11000 Belgrade, Serbia; 5 Clinic of Allergy and Immunology, University Clinical Center of Serbia, Koste Todorovica 2, 11000 Belgrade, Serbia

**Keywords:** autophagy, inflammation, COVID-19, p62, NSP, ORF3a

## Abstract

As autophagy can promote or inhibit inflammation, we examined autophagy-inflammation interplay in COVID-19. Autophagy markers in the blood of 19 control subjects and 26 COVID-19 patients at hospital admission and one week later were measured by ELISA, while cytokine levels were examined by flow cytometric bead immunoassay. The antiviral IFN-α and proinflammatory TNF, IL-6, IL-8, IL-17, IL-33, and IFN-γ were elevated in COVID-19 patients at both time points, while IL-10 and IL-1β were increased at admission and one week later, respectively. Autophagy markers LC3 and ATG5 were unaltered in COVID-19. In contrast, the concentration of autophagic cargo receptor p62 was significantly lower and positively correlated with TNF, IL-10, IL-17, and IL-33 at hospital admission, returning to normal levels after one week. The expression of SARS-CoV-2 proteins NSP5 or ORF3a in THP-1 monocytes caused an autophagy-independent decrease or autophagy-inhibition-dependent increase, respectively, of intracellular/secreted p62, as confirmed by immunoblot/ELISA. This was associated with an NSP5-mediated decrease in TNF/IL-10 mRNA and an ORF3a-mediated increase in TNF/IL-1β/IL-6/IL-10/IL-33 mRNA levels. A genetic knockdown of p62 mimicked the immunosuppressive effect of NSP5, and a p62 increase in autophagy-deficient cells mirrored the immunostimulatory action of ORF3a. In conclusion, the proinflammatory autophagy receptor p62 is reduced inacute COVID-19, and the balance between autophagy-independent decrease and autophagy blockade-dependent increase of p62 levels could affect SARS-CoV-induced inflammation.

## 1. Introduction

Coronavirus disease 2019 (COVID-19) is an ongoing pandemic disease caused by severe acute respiratory syndrome coronavirus 2 (SARS-CoV-2), which infects respiratory epithelium, vascular endothelial cells, and macrophages [1,2]. Patients range from asymptomatic or having a mild upper respiratory illness to severe viral pneumonia requiring hospitalization, which may progress to acute respiratory distress syndrome, multiorgan failure, and death. Innate immunity is crucial for the elimination of SARS-CoV-2, the removal of dead cells, and restoring tissue function, as well as coordinating/sustaining the protective adaptive immune responses of virus-specific T and B lymphocytes [3,4]. However, a dysregulated antiviral immune response and ensuing positive feedback between hyperinflammation and hypercoagulation contribute to the damage of the lungs and other organs in severe COVID-19 [5].

Macroautophagy (hereafter autophagy) is a fundamental homeostatic mechanism involving the sequestration of cytoplasmic material into vesicles called autophagosomes, and its subsequent delivery to lysosomes for degradation [6]. Autophagy is controlled by the expression and activity of autophagy-related (ATG) proteins, which interact in a time- and space-dependent manner to execute the autophagic program [7]. The key autophagy checkpoints include the beclin-1 (ATG6)-dependent initiation of autophagosome formation, ATG5-dependent lipidation of microtubule-associated protein 1 light chain 3 (LC3/ATG8) and subsequent autophagosome expansion, and the delivery of ubiquitinated intracellular cargo to autophagosomes by autophagy receptors such as p62/sequestosome 1 [6,7]. Autophagosomes then fuse with lysosomes, where autophagic cargo, including LC3 and p62, is eventually degraded. In addition to preserving cellular homeostasis by removing damaged proteins and providing energy and building blocks in stressful conditions, autophagy contributes to antiviral defense by directly destroying viruses in the autolysosomes [8]. On the other hand, some viruses, including SARS-CoV-2, can use double-membrane autophagosomes as platforms for their replication and protection from host defense mechanisms [9,10]. SARS-CoV-2 has been shown to suppress autophagic turnover (flux) by blocking the expansion of autophagosomes, their fusion with lysosomes, and/or lysosome acidification [11,12,13,14,15,16], thus evading degradation while using the accumulated autophagic structures for replication.

There is a complex interaction between autophagy and virus-induced inflammation. Type I interferons (IFN) stimulate autophagy [17], which in turn promotes the production of antiviral IFNs and proinflammatory mediators, as well as antigen presentation to T cells [18]. Indeed, autophagy induced by SARS-CoV-2 spike pseudovirions or SARS-CoV-2 GU-rich RNA was shown to stimulate the production of proinflammatory cytokines tumor necrosis factor (TNF), interleukin (IL)-1β, and IL-6 in human bronchial cells, epithelial kidney cells, and macrophages [19,20]. In addition, the increase in beclin-1 serum levels was associated with severe inflammation in COVID-19 [21], and the suppression of autophagy in leukocytes of severely ill COVID-19 patients correlated with impaired antigen presentation and T cell activation [22]. On the other hand, autophagy can also suppress antiviral immunity and inflammation [18], which may lead to viral evasion of immune response early during infection, but also limit tissue damage associated with prolonged/excessive inflammation. Accordingly, autophagy induced by SARS-CoV-2 matrix/membrane (M) protein, open reading frame 10 (ORF10), and non-structural protein 13 (NSP13) suppressed antiviral type I IFN production in kidney or cervical cancer cells [23,24], while ORF8-triggered autophagy degraded the MHC I molecules of SARS-CoV-2-infected kidney cells, thus preventing their lysis by cytotoxic T lymphocytes [25]. Consistent with the anti-inflammatory role of autophagy, its suppression in spleen macrophages or blood leukocytes was associated with hyperinflammation in severe COVID-19 [22,26].

In the present study, we combined the ex vivo and in vitro approaches to further examine the connection between inflammatory mediators and autophagy markers LC3, ATG5, and p62 in COVID-19. Our results demonstrate that the serum levels of p62 in COVID-19 patients at hospital admission were significantly reduced compared to healthy controls, as well as positively correlated with the levels of proinflammatory cytokines. Moreover, our in vitro data indicate that SARS-CoV-2 proteins ORF3a and NSP5 regulate the expression of proinflammatory cytokines through the modulation of p62 levels.

## 2. Materials and Methods

### 2.1. Patients and Controls

Blood samples from 26 COVID-19 patients (12 males and 14 females, median age 59.5 years, min–max 22–84 years) and 19 healthy volunteers (9 males and 10 females, median age 52 years, min–max 25–84 years) were collected at the University Clinical Center of Serbia (Belgrade, Serbia) in the period from 17 July to 17 August 2020. COVID-19 was confirmed by RT-PCR from a nasal/throat swab (18/26) or serology tests (8/26), while pneumonia was present in 92% of patients (24/26). Only the patients and controls who did not receive systemic immunomodulatory, anti-inflammatory, or chemotherapy in the last 6 months before sampling were included in the study. The patient samples were collected during regular diagnostic testing at admission and one week after receiving COVID-19 therapy, which included antibiotics/anticoagulants (100% of patients), hydroxychloroquine (89%), oxygen (54%), corticosteroids (42%), favipiravir (19%), and tocilizumab (15%). The seven-day time point (approx. 10 days from the appearance of symptoms) was chosen as the key moment in the clinical progression of COVID-19 to a more severe form when immune dysregulation occurs [27]. The Charlson Comorbidity Index (CCI), Modified Early Warning Score (MEWS) for clinical deterioration, and Pneumonia Severity Index (PSI) were calculated as previously described [28,29,30]. Based on the WHO recommendations (https://www.who.int/publications/i/item/WHO-2019-nCoV-clinical-2022.2, accessed on 15 November 2022), the disease with no signs of severe pneumonia and no need for oxygen therapy (SpO_2_ > 93%) was considered moderate, respiratory rate > 30 acts/min and need for oxygen therapy (2–5 L/min) were criteria for severe disease, while patients requiring high oxygen supply (>5 L/min) or assisted/mechanical ventilation were considered critically ill. The study was approved by the Ethical Committee of the University Clinical Centre of Serbia and the Faculty of Medicine, University of Belgrade. All participants signed the informed consent in accordance with the Declaration of Helsinki.

### 2.2. Blood Plasma Isolation

Venous blood was collected into 8 mL Vacutainer lithium heparin plasma separator tubes (BD Biosciences, San Diego, CA, USA) and centrifuged at 2000× *g* (15 min, 4 °C) to obtain plasma. The plasma samples were transferred to 2 mL cryogenic tubes (Thermo Fisher Scientific, Waltham, MA, USA) and frozen at −80 °C for the measurement of cytokines and autophagy markers.

### 2.3. Cytokine Quantification by Flow Cytometry

The plasma concentration of twelve cytokines (IFN-α2, IFN-γ, TNF, IL-1β, IL-6, IL-8, IL-10, IL-12p70, IL-17A, IL-18, IL-23, and IL-33) was measured by flow cytometry using a multiplex bead-based assay (#740809, LEGENDplex™; Biolegend Inc., San Diego, CA, USA), according to the manufacturer’s instructions. Briefly, standards and plasma samples were incubated with capture beads in polypropylene 96-well V-bottom plates for 2 h. After washing the plate, biotinylated detection antibodies were added to each well and incubated for 1 h. Streptavidin-phycoerythrin (SA-PE) was subsequently added and incubated for 30 min. The plate was washed, and samples were resuspended in wash buffer and transferred to 5 mL FACS tubes for analysis (BD Biosciences, San Diego, CA, USA). Sample acquisition was performed on a FACSAriaIII flow cytometer using FACSDiva 6.0 software (BD Biosciences, San Diego, CA, USA), and the data were analyzed with LEGENDplex^TM^ Data Analysis Software. Cytokines were identified based on the bead size, their intrinsic fluorescence, and the fluorescent signal emitted from the anti-cytokine antibody/SA-PE complex. Cytokine concentration in samples was determined from the geometric mean fluorescence intensity of PE interpolated on the standard curves calculated from eight standard dilutions measured in duplicate.

### 2.4. Quantification of Autophagy Markers by Enzyme-Linked Immunosorbent Assay (ELISA)

Autophagy markers in the plasma or cell culture supernatants were measured using commercial ELISA kits for the detection of p62 (ab289654), LC3A (ab239432), beclin-1 (ab254511) (all from Abcam, Cambridge, UK), and Atg5 (ABIN6965047; Antibodies-Online GmbH, Aachen, Germany), according to the manufacturer’s instructions. The lower detection limits, intra-assay, and inter-assay variability coefficients provided by the manufacturer were, respectively, 0.1 ng/mL, 3.2%, and 5.7% (p62); 2.1 pg/mL, 3.6%, and 3.5% (LC3); 0.7 ng/mL, 5.2%, and 1.4% (beclin-1); and 0.2 ng/mL, <10%, and <12% (ATG5).

### 2.5. THP-1 Cell Culture

Human acute monocytic leukemia THP-1 cells (Elabscience, Houston, TX, USA) were maintained at 37 °C in a humidified atmosphere with 5% CO_2_ in a HEPES (10 mM)-buffered RPMI 1640 cell culture medium with 2 mM L-glutamine, supplemented with 5% fetal bovine serum (FBS), 1 mM sodium pyruvate, 1% non-essential amino acids, 0.05 mM β-mercaptoethanol, and 1% antibiotic/antimycotic mixture (all from Capricorn Scientific GmbH, Ebsdorfergrund, Germany). For the experiments, cells were incubated in 12-well plates (1 × 10^6^ cells/well) in 2 mL of cell culture medium, in the presence or absence of lysosomal inhibitor bafilomycin A1 (50 nM; Merck, Darmstadt, Germany) or Toll-like receptor (TLR)7/8 agonist resiquimod (R848) (10 µM; InvivoGen, Toulouse, France), as described in the figure legends. Dimethyl sulfoxide was used as a vehicle for both bafilomycin A1 and R848, and its amount in the cell cultures (including untreated controls) was 0.01%.

### 2.6. Expression of SARS-CoV-2 Proteins in THP-1 Cells

Plasmids encoding SARS-CoV-2 proteins NSP5 (#152344) and ORF3A (#152341) were provided from Addgene (Cambridge, MA, USA). Empty control plasmid pTwist-CMV-Puro 6 was generated by double restriction digestion of NSP4 pTwist-CMV-Puro 6 with HindIII (FD0596) and PvuII (FD0634) in FastDigest Buffer (B64) (all from Thermo Fisher Scientific, Waltham, MA, USA) at 37 °C for 30 min, and then blunted with Klenow fragment (M0212; New England BioLabs, Ipswich, MA, USA) and ligated with T4 ligase (EL0011; ThermoFisher Scientific, Waltham, MA, USA) at 15 °C for 9 h, as recommended in Addgene protocol. After multiplication in DH5α competent *E. coli*, the plasmids were isolated using GeneJET Plasmid Maxiprep Kit (K0491; ThermoFisher Scientific, Waltham, MA, USA) according to the manufacturer’s protocol. Empty plasmid and plasmids encoding SARS-CoV-2 proteins (2 µg of plasmids in 100 µL of transfection buffer) were transfected by electroporation into THP-1 cells using the SG Cell Line 4D-Nucleofector V Kit and 4D-Nucleofector (Lonza, Basel, Switzerland), according to the manufacturer’s instructions. After transfection, cells were transferred to 12-well plates (1 × 10^6^ cells/well) and incubated in a complete cell culture medium with 5% FBS as described in the figure legends.

### 2.7. RNA Interference

THP-1 cells were electroporated with small interfering RNA (siRNA) against human p62 (sc-29679), LC3B (sc-43390), or corresponding control siRNA (sc-37007) (all from Santa Cruz Biotechnology, Santa Cruz, CA, USA), using SF Cell Line 4D-Nucleofector X Solution and program DV-100 on a 4D-Nucleofector (Lonza, Basel, Switzerland) following the manufacturer’s instructions. After transfection, cells were transferred to 12-well plates (1 × 10^6^ cells/well) and incubated in a complete cell culture medium with 5% FBS, as described in the figure legends.

### 2.8. Immunoblotting

Cells were lysed in RIPA buffer (50 mM Tris, pH 8.0, 150 mM NaCl, 1% IGEPAL CA-630, 0.5% sodium deoxycholate, 0.1% SDS, and protease/phosphatase inhibitor cocktail, all from Merck, Darmstadt, Germany), stored on ice for 30 min, centrifuged at 14,000× *g* for 15 min at 4 °C, and the supernatants were collected. Equal protein amounts from each sample (10 µg for p62 and LC3, 20 µg for NSP5, and 50 µg for FLAG-ORF3a) were separated by sodium dodecyl sulfate-polyacrylamide gel electrophoresis and transferred to nitrocellulose membranes (Bio-Rad, Hercules, CA, USA). Rabbit anti-human antibodies against p62 (NBP-1-48320; Novus Biologicals, Littleton, CO, USA), NSP5 (#51661), LC3B (#2775), and actin (#4968) as a loading control, as well as rabbit anti-FLAG (#14793) for the detection of ORF3a (all from Cell Signaling Technology, Cambridge, MA, USA) were used as primary antibodies. The peroxidase-conjugated goat anti-rabbit IgG (11-035-144; Jackson ImmunoResearch, West Grove, PA, USA) was used as a secondary antibody, and specific protein bands were visualized by enhanced chemiluminescence using ChemiDoc MP Imaging System (Bio-Rad, Hercules, CA, USA). The intensity of protein bands was measured by densitometry using Image Lab software (Bio-Rad, Hercules, CA, USA).

### 2.9. Reverse Transcription-Quantitative Polymerase Chain Reaction (RT-qPCR)

RNA was extracted from THP-1 cells using GeneJET RNA Purification Kit (Thermo Fisher Scientific, Waltham, MA, USA) and reverse transcribed with MuLV reverse transcriptase and random hexamers (Thermo Fisher Scientific, Waltham, MA, USA), following the manufacturer’s recommendations. RT-qPCR was performed in a Mastercycler Realplex^2^ (Eppendorf, Hamburg, Germany) using MicroAmp Optical 96-well reaction plates, TaqMan Universal PCR Master Mix, and TaqMan primers/probes (all from Thermo Fisher Scientific, Waltham, MA, USA) for human *p62/SQSTM1* (Hs00177654_m1), *TNF* (Hs00174128_m1), *IL-1β* (Hs01555410_m1), *IL-6* (Hs00174131_m1), *IL-10* (Hs00961622_m1), *IL-33* (Hs01125943_m1), and 18S ribosomal RNA (*RN18S*; Hs03928985_g1), TATA-box-binding protein (*TBP*; Hs00427620_m1), and hypoxanthine-guanine phosphoribosyltransferase 1 (*HPRT1*; Hs02800695_m1) as housekeeping genes. Assays were performed in duplicates using the thermal cycling conditions recommended by the manufacturer. The geomean cycle of threshold (Ct) values of *RN18S/TBP*/*HPRT1* genes were subtracted from the Ct values of target genes to obtain ΔCt. The ΔΔCt values were obtained by subtracting the ΔCt values of control samples (cells transfected with control plasmid/siRNA) from the ΔCt values of SARS-CoV-2 plasmid/p62 siRNA-transfected cells, and the relative gene expression was calculated as 2^−ΔΔCt^.

### 2.10. Statistical Analysis

Since the Wilks–Shapiro test indicated that the concentrations of most cytokines and autophagy markers were not normally distributed, the differences in their levels were analyzed using a two-tailed Mann–Whitney U test for comparing control subjects and COVID-19 patients, or two-tailed Wilcoxon signed-rank test for comparing COVID-19 patients before and after therapy (the data were presented as median values and interquartile range). The Spearman correlation test was employed to assess the correlation between different parameters (the linear trendlines in correlation graphs are only for visualization of correlation trends and do not reflect the exact relationship between variables). Receiver Operating Characteristic (ROC) curve analysis was used to assess the predictive value of different parameters, and the cut-off points were determined by calculating the Youden index as the maximum value of (sensitivity + specificity − 1) [31]. The statistical significance of the differences in the in vitro experiments was assessed using a two-tailed one-sample *t*-test or paired *t*-test where appropriate, as recommended for very small sample sizes [32], following log_2_ transformation of the data. The *p* values < 0.05 were considered significant. To reduce the risk of missing the true effects, no corrections for multiple comparisons were made [33]. All statistical analyses were performed using SPSS 22.0 software (IBM SPSS, Chicago, IL, USA).

## 3. Results

### 3.1. Clinical Characteristics of COVID-19 Patients

The COVID-19 and control group did not significantly differ in sex distribution (male/female 12/14 vs. 9/10, *p* = 1.000, Fisher’s exact test) or age (median/interquartile range 59/47-72 vs. 52/44-66 years, *p* = 0.448, Mann–Whitney U test). The main clinical features of COVID-19 patients are given in Supplementary Table S1. Comorbidities were present in 17 patients and included cardiovascular disease, hypertension, hyperlipidemia, type 2 diabetes, asthma, chronic obstructive pulmonary disease, and history of malignancy, with nine patients having had more than one comorbidity (median CCI/interquartile range values 1.5/0–5). The most frequent symptoms of COVID-19 were fever, cough, dyspnea, and diarrhea, and all but two patients had radiologically confirmed pneumonia. All patients received antibiotic/anticoagulant therapy, while 88.5% of patients were additionally treated with hydroxychloroquine, 53.8% with oxygen, 42.3% with corticosteroids, 19.2% with favipiravir, and 15.5% with tocilizumab. All patients with moderate and severe disease, as well as two of six critically ill patients completely recovered after 4 weeks, three patients were transferred to the intensive care unit, and one patient died. The main hematological and biochemical features of COVID-19 patients, as well as the values of MEWS and PSI at admission and one week after, are presented in Supplementary Table S2. A total of 8 patients had leukopenia (<4.3 × 10^9^/L), 11 patients had lymphopenia (<1.1 × 10^9^/L), 5 patients had thrombocytopenia (<140 × 10^9^/L), and 21 patients had increased blood C-reactive protein (CRP) levels (>5.0 mg/mL). The numbers of total leukocytes, lymphocytes, and thrombocytes were significantly increased, while the CRP levels were reduced one week after diagnosis, indicating the improvement of lymphopenia and thrombocytopenia, and a decrease in inflammation. There were no significant changes in neutrophil, monocyte, and erythrocyte counts, or hemoglobin levels. MEWS and PSI values, reflecting the overall clinical status of the patients and the severity of pneumonia, respectively, were significantly reduced one week after admission, mirroring the clinical recovery of patients. The ROC curve analysis demonstrated that high CCI, MEWS, and PSI scores, but not CRP values, were good predictors of poor disease outcome, defined as no clinical recovery after 4 weeks (Supplementary Figure S1).

### 3.2. The Levels of Proinflammatory Cytokines Are Increased in COVID-19 Patients

**Figure 1:**
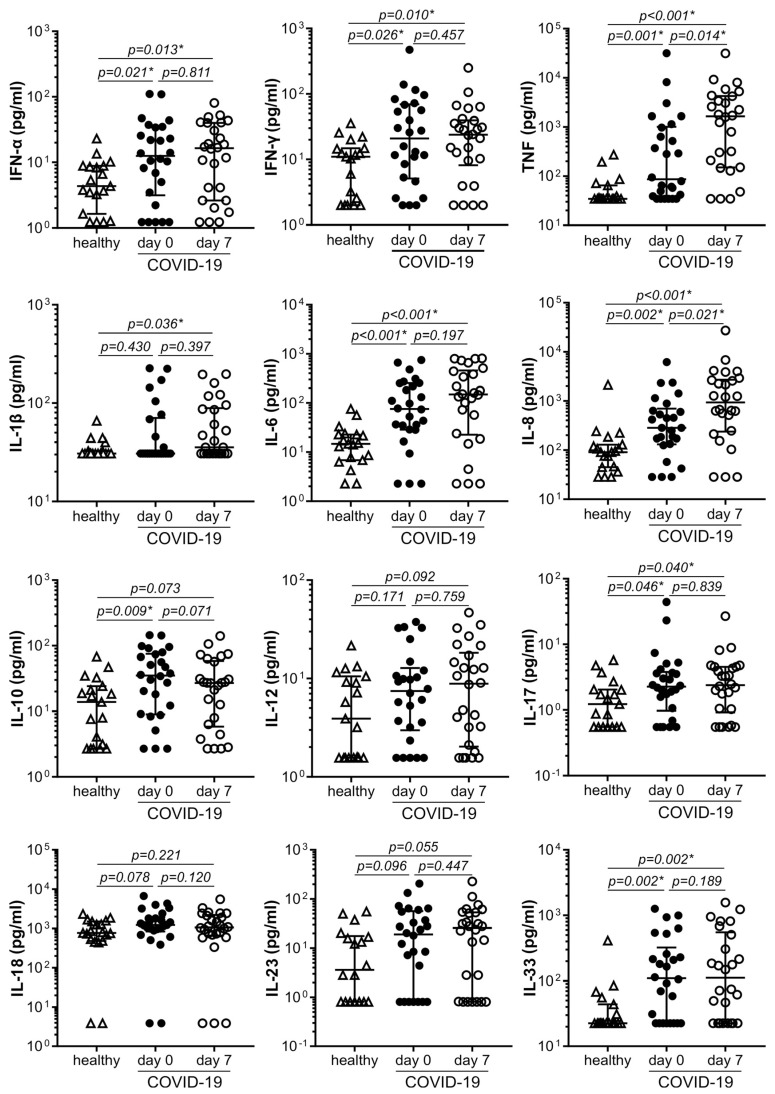
Cytokine levels are increased in COVID-19. Cytokine levels in the blood plasma of control subjects (n = 19) and COVID-19 patients (n = 26) at hospital admission (day 0) and 7 days after therapy were determined by a multiplex flow cytometry bead assay (median and 25/75 percentile values are shown as lines; * *p* < 0.05, two-tailed Mann–Whitney U test or Wilcoxon signed-rank test for controls vs. COVID-19 patients or COVID-19 patients at day 0 vs. day 7, respectively).

The blood concentrations of the antiviral cytokine IFN-α and proinflammatory cytokines TNF, IFN-γ, IL-6, IL-8, IL-17, and IL-33 were significantly increased in COVID-19 patients both at admission and one week later, compared to healthy controls (Figure 1). TNF and IL-8 in COVID-19 patients were additionally increased after one week compared to the levels at admission (Figure 1). The levels of the immunoregulatory cytokine IL-10 were elevated only at admission, while proinflammatory IL-1β was increased only one week later (Figure 1). The concentrations of the proinflammatory IL-12, IL-18, and IL-23 were not significantly different in COVID-19 patients compared to healthy controls, or between COVID-19 patients before and after one-week therapy (Figure 1). The presence of comorbidities did not contribute to the increase in cytokine levels in COVID-19, as there was no correlation between CCI and cytokine concentrations (Supplementary Figure S2). Among the cytokines that were increased in COVID-19 patients at admission, only IL-6 displayed a positive correlation with MEWS as a measure of disease severity (Supplementary Figure S3), while no significant correlation was observed between cytokine levels and PSI (Supplementary Figure S4). Moreover, the cytokine levels could not successfully predict the disease outcome at 4 weeks (Supplementary Figure S5). Therefore, the production of proinflammatory cytokines was increased in COVID-19 but did not correlate with disease severity (except for IL-6) or outcome.

### 3.3. The Concentration of p62 Is Reduced in COVID-19 and Correlates with Cytokine Production

**Figure 2:**
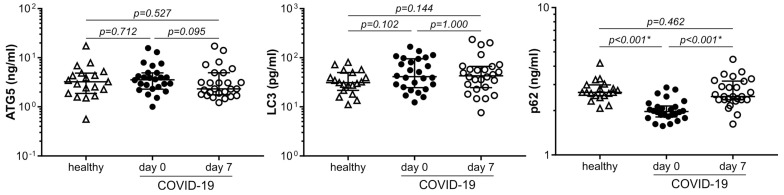
p62 levels are reduced in COVID-19. The levels of autophagy markers p62, LC3, and ATG5 in the blood plasma of control subjects (n = 19) and COVID-19 patients (n = 26) at hospital admission (day 0) and 7 days later were determined by ELISA (median and 25/75 percentile values are shown as lines; * *p* < 0.05, two-tailed Mann–Whitney U test or Wilcoxon signed-rank test for controls vs. COVID-19 patients or COVID-19 patients at day 0 vs. day 7, respectively).

**Figure 3:**
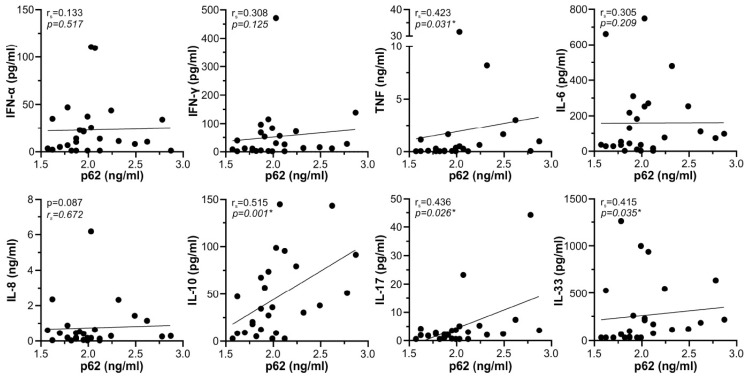
Correlation between p62 and cytokine levels in COVID-19. The correlation between blood plasma levels of p62 and cytokines in COVID-19 patients (n = 26) at hospital admission was assessed by the Spearman rank-order test (r_s_-Spearman’s correlation coefficient; * *p* < 0.05).

We next assessed the levels of key autophagy markers p62, LC3, ATG5, and beclin-1 in the blood of healthy controls and COVID-19 patients at admission and one week later. All autophagy markers except beclin-1 (not shown) were detectable in all three sample groups. Moreover, their levels were not correlated with those of cellular damage marker LDH (Supplementary Figure S6), indicating that the presence of autophagy markers in the circulation was not simply a consequence of their release from damaged cells. The three sample groups did not significantly differ in the blood levels of LC3 or ATG5 (Figure 2). On the other hand, the blood concentration of p62 was significantly reduced in COVID-19 patients at admission compared to healthy controls, returning to levels comparable to those of healthy subjects one week later (Figure 2). Moreover, ROC curve analysis showed that low p62 at admission was able to discriminate COVID-19 patients from healthy controls with 89% sensitivity and 95% specificity at the cut-off level of 2.14 ng/mL, performing better than high cytokine levels in that respect (Supplementary Figure S7). The observed decrease in p62 was not related to the presence of comorbidities, as p62 levels did not correlate with CCI values (Supplementary Figure S8). Additionally, p62 levels did not correlate with MEWS or PSI, and could not predict disease outcome (Supplementary Figure S8). On the other hand, there was a significant positive correlation between the levels of p62 at admission and TNF, IL-10, IL-17, or IL-33 (Figure 3). These results indicate a link between the circulating p62 and inflammatory response in COVID-19.

### 3.4. In Vitro Modulation of p62 and Autophagy by ORF3a and NSP5

**Figure 4:**
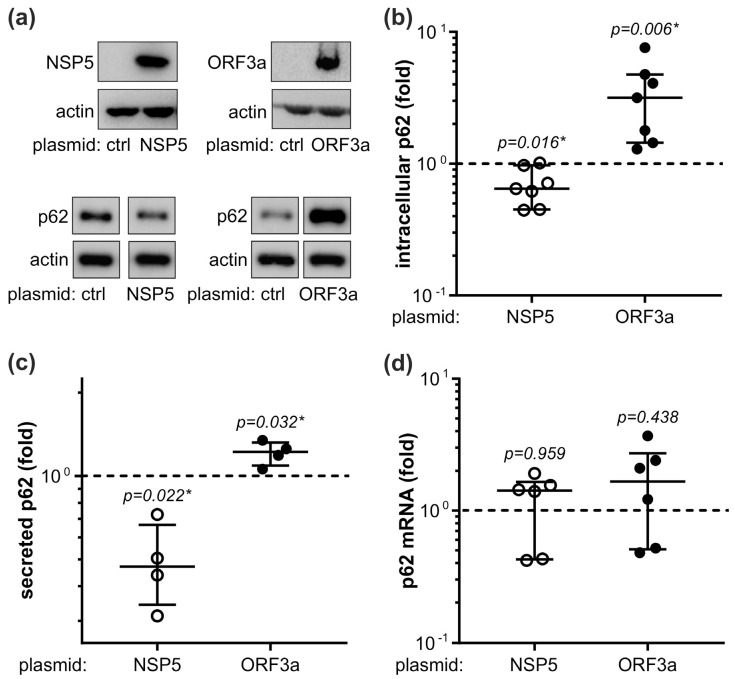
NSP5 and ORF3a modulate p62 levels in THP-1 cells. (**a**–**d**) THP-1 cells were transfected with control plasmid or plasmids encoding NSP5 or ORF3a, and incubated in a cell culture medium with 5% FBS. After 48 h, intracellular protein levels of NSP5, ORF3a, and p62 were analyzed by immunoblotting (**a**), and p62 levels were quantified by densitometry (**b**). The concentration of p62 in cell culture supernatants was determined by ELISA (**c**), and p62 mRNA levels were measured by RT-qPCR (**d**). Representative blots are shown in (**a**), while the data from seven (**b**), four (**c**), or six (**d**) independent experiments are presented as fold change relative to the control value (dashed line) obtained in cells transfected with the control plasmid (median and 25/75 percentile values are shown as lines; * *p* < 0.05, two-tailed one-sample *t*-test). Original blots from (**a**) are shown in Supplementary Figure S10.

**Figure 5:**
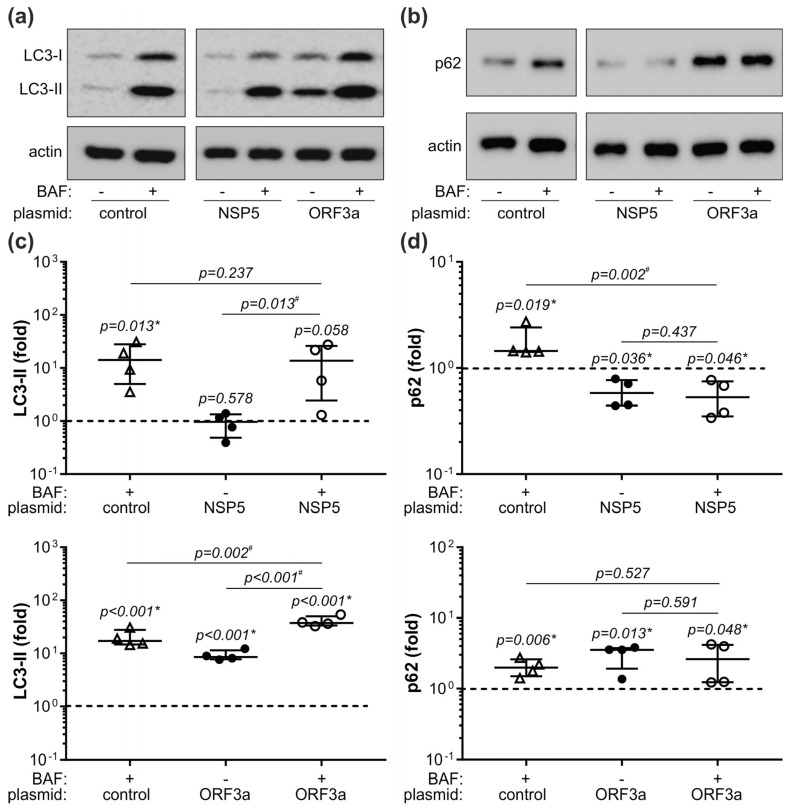
Autophagy role in NSP5/ORF3a-mediated p62 modulation. (**a**–**d**) THP-1 transfected with control plasmid or plasmids encoding NSP5/ORF3a were incubated in cell culture medium with 5% FBS, in the presence or absence of lysosomal inhibitor bafilomycin A1 (BAF; 50 nm) during the last 6 h of 48 h incubation period. Intracellular LC3-II and p62 were detected by immunoblotting (**a**,**b**), and their levels were quantified by densitometry and expressed relative to β-actin as a loading control (**c**,**d**). Representative blots are shown in (**a**,**b**), while the data from four independent experiments (**c**,**d**) are presented as fold change relative to the control value (dashed line) obtained in cells transfected with control plasmid and not treated with BAF (median and 25/75 percentile values are shown as lines; * *p* < 0.05, two-tailed one-sample *t*-test vs. untreated control; # *p* < 0.05, two-tailed paired *t*-test). Original blots from (**a**,**b**) are shown in Supplementary Figure S11.

We next used an in vitro approach to further assess the interplay between the expression of p62, autophagy, and inflammatory mediators in SARS-CoV-2 infection. In preliminary experiments we screened various SARS-CoV-2 proteins for their ability to affect p62 levels in human monocytic leukemia cell line THP-1. NSP5, the main SARS-CoV-2 protease involved in the formation of the viral replicase complex [34], and ORF3a, the accessory protein essential for SARS-CoV-2 replication and release [35], were selected as the two viral proteins whose expression significantly decreased and increased, respectively, the intracellular levels of p62 in THP-1 cells (Figure 4a,b). This was mirrored by the decrease or increase in p62 levels in the supernatants of THP-1 cells expressing NSP5 or ORF3a, respectively (Figure 4c). Neither NSP5 nor ORF3a affected the expression of p62 mRNA in THP-1 cells (Figure 4d), indicating that p62 modulation occurred at the post-transcriptional level. To evaluate if NSP5 or ORF3a altered p62 degradation in lysosomes, we assessed autophagic flux by measuring the amounts of p62 and LC3-I lipidation product LC3-II in the presence of lysosomal inhibitor bafilomycin A1 [36]. Expectedly, the inhibition of lysosomal proteolysis by bafilomycin A1, limited to the last 6 h of incubation to avoid its cytotoxicity [36], significantly increased the intracellular levels of both LC3-II and p62 (Figure 5a,b). The levels of LC3-II precursor LC3-I were also increased by bafilomycin A1 both in control and NSP5/ORF3a-expressing cells (Supplementary Figure S9), possibly due to negative feedback inhibition of LC3-I/LC3-II conversion. NSP5 expression alone did not change the levels of autophagosome marker LC3-II and was unable to alter its increase by bafilomycin A1, indicating that NSP5 did not modulate autophagic flux (Figure 5a). Accordingly, p62 downregulation by NSP5 was preserved in the presence of bafilomycin A1 (Figure 5b), thus confirming that the NSP5-mediated decrease in p62 was independent ofits autophagic turnover. On the other hand, ORF3a significantly increased LC3-II levels both in the absence and presence of bafilomycin, which is consistent with the induction of autophagosome formation (Figure 5a). However, no additional increase in p62 levels was observed in ORF3a-expressing cells treated with bafilomycin A1 (Figure 5b), suggesting that ORF3a increased p62 levels by blocking its lysosomal degradation. Collectively, these data indicate that NSP5 decreased p62 levels without affecting autophagic flux, while the ORF3a-mediated increase in p62 was associated with the induction of incomplete autophagy and blockade of autophagic flux.

### 3.5. NSP5 and ORF3a Regulate Cytokine Expression through p62 Modulation

**Figure 6:**
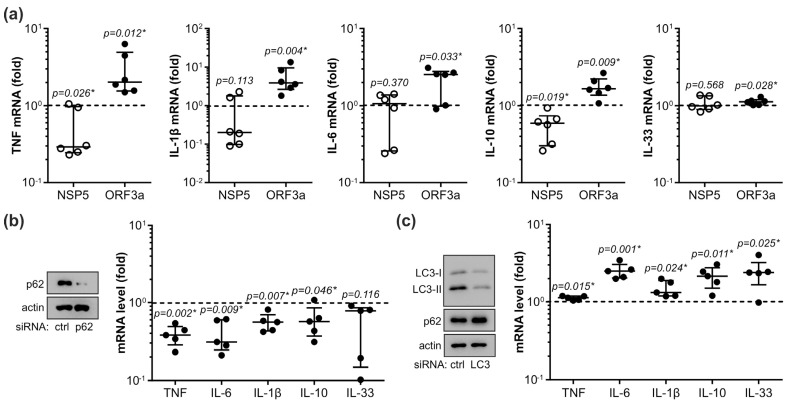
NSP5 and ORF3a regulate cytokine expression through p62. (**a**–**c**) THP-1 cells were transfected with control plasmid or plasmids encoding NSP5/ORF3a (**a**), control/p62 siRNA (**b**), or control/LC3 siRNA (**c**). After 48 h, the cytokine (TNF, IL-1β, IL-6, IL-10, IL-33) mRNA levels were measured by RT-qPCR (**a**–**c**). The downregulation of p62 and LC3-II was confirmed by immunoblotting (**b**,**c**). The data from six (**a**) or five (**b**,**c**) independent experiments are presented as fold change relative to the control value (dashed line) obtained in cells transfected with control plasmid/siRNA (median and 25/75 percentile values are shown as lines; * *p* < 0.05, two-tailed one-sample *t*-test). Original blots from (**b**,**c**) are shown in Supplementary Figure S13.

Finally, we examined the possibility that NSP5 and ORF3a might influence cytokine expression through the modulation of p62. The downregulation of p62 by NSP5 was associated with the decrease in TNF and IL-10 mRNA levels, and the ORF3a-mediated p62 increase was accompanied by a significantly higher expression of TNF, IL-1β, IL-6, IL-10, and IL-33 mRNA in THP-1 cells (Figure 6a). A similar pattern, but with less pronounced differences, was observed when cytokine mRNA expression was stimulated with R848, an agonist of ssRNA-recognizing TLR7/8 (Supplementary Figure S12). The RNA interference-mediated p62 knockdown reduced the mRNA expression of TNF, IL-6, IL-1β, and IL-10 (Figure 6b), while autophagy suppression by LC3 siRNA increased the intracellular levels of p62 protein and mRNA of all tested cytokines (Figure 6c), thus mimicking the effects of NSP5 and ORF3a, respectively. These data indicate that ORF3a and NSP5 might affect cytokine expression in THP-1 cells through the modulation of p62 levels.

## 4. Discussion

**Figure 7:**
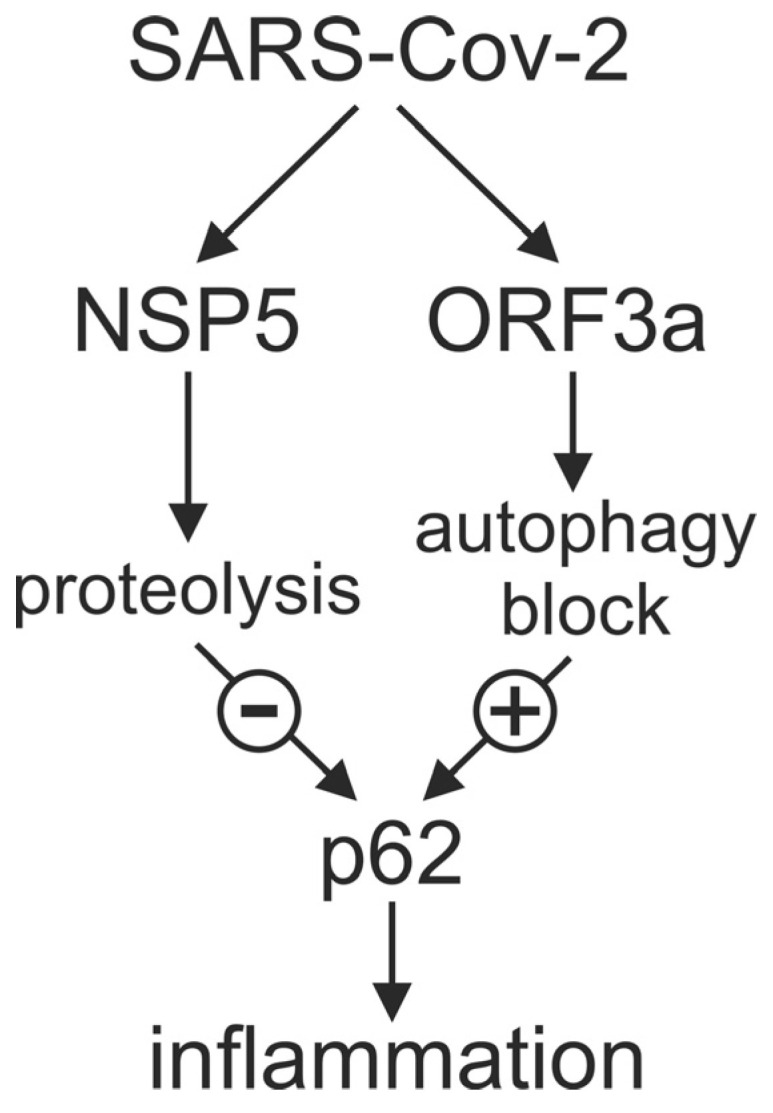
NSP5 and ORF3a of SARS-CoV-2 modulate inflammation via autophagy receptor p62. NSP5 decreases and ORF3a increases the levels of proinflammatory p62 through proteolytic degradation and blockade of autophagic flux, respectively. The balance between these two actions might influence the overall effect of SARS-CoV-2 on inflammation in COVID-19.

The present study for the first time indicates a role for autophagic cargo receptor p62 in the regulation of inflammatory response in COVID-19. The blood levels of p62 were decreased but positively correlated with those of proinflammatory cytokines in COVID-19 patients. Moreover, SARS-CoV-2 proteins ORF3a and NSP5 regulated the in vitro expression of proinflammatory cytokines through autophagy-dependent and -independent modulation of p62 levels, respectively, thus further supporting the involvement of p62 in the modulation of COVID-19-associated inflammation (Figure 7).

Several studies so far have examined various autophagy markers in COVID-19. The blood levels of proautophagic beclin-1 were increased, while the expression of LC3 mRNA and LC3-I/II conversion in blood mononuclear cells were reduced in COVID-19 compared to control subjects [21,22]. On the other hand, the levels of both LC3-II and p62 in blood mononuclear cells incubated in vitro for 24 h, as well as p62 protein accumulation in spleen macrophages, were increased in COVID-19 patients [26,37]. However, the samples in these studies were collected from patients who received unspecified therapy [21,26,37] or therapy including autophagy inhibitor chloroquine [22]. Therefore, further analysis is required to resolve the apparent discrepancies and confirm if the observed changes in autophagy markers were related to SARS-CoV-2 infection itself, tissue/cell-specific effects, and/or the applied therapy. In addition, a recent study assessed COVID-19 patients for blood concentrations of p62 and LC3 without comparing them to healthy subjects [38]. To the best of our knowledge, our report is the first to show that the blood plasma levels of autophagy receptor p62 in newly diagnosed COVID-19 patients before therapy are significantly decreased compared to healthy controls. Importantly, the observed decline in circulating p62 was unrelated to associated comorbidities, indicating that it was a consequence of SARS-CoV-2 infection per se. Moreover, the absence of p62/LDH correlation suggests that systemic cell damage was not the major source of circulating p62 in COVID-19 patients, which is consistent with the findings that macrophages/monocytes and other cell types can actively secrete p62 [39,40,41,42,43]. While the blood levels of p62 returned to normal values after one week, it remains to be evaluated if this was due to the recovery from the disease or the therapy that included chloroquine, which increases p62 levels by blocking its degradation in autolysosomes [44]. Unlike p62, the circulating levels of LC3 and ATG5, both involved in autophagosome formation, were not significantly altered in COVID-19 patients in our study, although a tendency towards LC3 increase was observed.

It has been proposed that the secretion of autophagy cargo receptors, including p62, might serve as a buffering mechanism to control the intracellular accumulation of these important effector proteins [43]. Our in vitro experiments with ORF3a/NSP5-expressing cells support this assumption by demonstrating the correlation between the amounts of intracellular and secreted p62. With this in mind and knowing that p62 is selectively degraded during autophagy [45], one might be tempted to speculate that the observed decrease in circulating p62 in COVID-19 could reflect a systemic induction of autophagic flux and subsequent decline in intracellular p62 levels. Indeed, the expression of lysosomal genes was upregulated in blood mononuclear cells of COVID-19 patients [38], while ORF10 and NSP13 of SARS-CoV-2 were reported to suppress type I IFN expression by autophagic degradation of mitochondrial antiviral signaling protein and TANK-binding kinase 1, respectively [23,24]. On the other hand, NSP6, ORF7a, and particularly ORF3a have been shown to block autophagic turnover by preventing autophagosome-lysosome fusion and/or by reducing the acidity of lysosomes [13,14,15,46,47,48]. The current consensus, supported by several in vitro and in vivo studies, is that SARS-CoV-2 induces autophagy but blocks its completion, using accumulated autophagosomes as replication platforms [11,12,49,50]. This is consistent with our in vitro data showing that ORF3a increases both LC3-I/II conversion and p62 levels, reflecting an induction of autophagosome formation and suppression of autophagic flux, respectively. On the other hand, SARS-CoV-2 NSP5 efficiently decreased the intracellular p62 levels even when autophagic degradation was blocked by lysosomal inhibition, which is consistent with its recently reported ability to directly cleave p62 at glutamic acid 354 [51]. Collectively, these findings indicate that NSP5-mediated p62 degradation, rather than the increase in autophagic flux, might be involved in the observed decrease in p62 blood levels in COVID-19 patients. While this agrees with the p62 decrease in SARS-CoV-2-infected lung adenocarcinoma Calu3 cells [24], it should be noted that SARS-CoV-2 infection did not affect or even increased p62 levels in monkey kidney epithelial cell line Vero E6 and cervical cancer cell line HeLa [49,52,53,54,55]. Therefore, the balance between ORF3a-mediated p62 increase and NSP5-mediated p62 degradation, affecting the overall effect of SARS-CoV-2 infection on the levels of intracellular/secreted p62, might depend on the cell type and other factors such as viral load and infection duration.

Our data corroborate the previously reported increase in antiviral/proinflammatory cytokines IFN-α, TNF, IFN-γ IL-1, IL-6, IL-8, IL-17, and IL-33 in COVID-19 [56,57,58,59,60,61], confirming the association between high IL-6 levels and disease severity [61]. We also observed an increase in immunoregulatory IL-10, which could serve as a negative feedback signal to restrict hyper-inflammation [62], or, as recently proposed, contribute to inflammatory pathogenesis and severity of COVID-19 [61,63]. Interestingly, while most of the proinflammatory cytokines remained increased one week from diagnosis, CRP returned to normal levels. As IFN-α was found to suppress IL-6/IL-1ß-induced CRP gene transcription and protein production [64,65], it is conceivable that in COVID-19, high levels of antiviral IFN-α one week after diagnosis might contribute to CRP downregulation despite the prolonged increase in CRP-inducing proinflammatory cytokines. Although the p62 blood levels in COVID-19 patients at diagnosis were decreased, they were positively correlated with the amounts of TNF, IL-17, IL-10, and IL-33. Moreover, p62 modulation by NSP5 and ORF3a in vitro was involved in the regulation of TNF, IL-1, IL-6, IL-10, and IL-33 mRNA expression in THP-1 monocytic cell line, indicating a proinflammatory role of this autophagy receptor in COVID-19. Indeed, it has recently been shown in various experimental models that p62 can trigger the expression of proinflammatory cytokines through activation of the transcription factor nuclear factor-κB [39,66,67,68]. It remains to be investigated if a similar p62-dependent mechanism might be involved in the regulation of COVID-19-associated inflammation.

There is a question about the possible clinical relevance of the observed p62 downregulation in COVID-19. The p62 cleavage by NSP5 and subsequent block of selective autophagy of viral proteins [51], together with ORF3a-mediated autophagy suppression at the degradation stage [13,14,25,48] might enable SARS-CoV-2 to subvert cellular autophagic machinery and use it for its replication. Accordingly, a decrease in serum concentrations of p62 was associated with more severe disease in a subgroup of COVID-19 patients aged 50 or below [38]. However, we did not find any association between blood p62 levels and COVID-19 severity/outcome. This could be due to a relatively small group size, which also prevented the stratification of patients according to sex, age, or various clinical/biochemical characteristics. Moreover, the clinical significance of the positive correlation between p62 and proinflammatory cytokines in COVID-19 was somewhat difficult to interpret in the context of systemic p62 decrease. The downregulation of p62 by SARS-CoV-2 might promote viral replication by preventing an anti-viral inflammatory response. On the other hand, the increased p62 production in certain patient subgroups and/or stages of infection could participate in COVID-19-associated hyperinflammation. Interestingly, we have observed that the levels of most cytokines, except IL-10, remained high one week after therapy, while TNF and IL-8 were additionally increased compared to the levels at diagnosis. While one could speculate that the increase in p62 might contribute to maintaining high cytokine production in recovering patients, the exact mechanisms and clinical relevance of this phenomenon are still to be explored. Finally, ROC analysis revealed that low p62 levels discriminated COVID-19 patients from healthy subjects better than the increase in cytokine/CRP levels. Therefore, low p62 seems worthy of investigation as a potential COVID-19 marker when clinical signs of disease are accompanied by negative RT-PCR and/or antigen tests [69].

The limitations of the present study include a small sample size, the absence of autophagy/inflammation markers assessment in different blood cell types, and the use of the selective expression of viral proteins as a surrogate for virus infection. The analysis of large, stratified patient populations; various leukocyte subtypes; and cells infected with NSP5/ORF3a SARS-CoV-2 mutants would shed additional light on p62-inflammation interplay in COVID-19.

In conclusion, the present study indicates that the balance between the proinflammatory action of ORF-3a-mediated increase and anti-inflammatory action of NSP5-mediated degradation of autophagy receptor p62 might contribute to the overall effect of SARS-CoV-2 on the host inflammatory response. While the latter effect seems to prevail in acute COVID-19, future studies should explore the mechanisms and clinical significance of p62modulation and its immunoregulatory role in various forms of this disease.

## Data Availability

The data presented in this study are available in the article and supplementary material.

## Supplementary Materials

The following supporting information can be downloaded at: https://www.mdpi.com/article/10.3390/cells12091282/s1, Figure S1: The ability of various parameters to predict COVID-19 outcome; Figure S2: Correlation between cytokine levels and comorbidities in COVID-19; Figure S3: Correlation between cytokine levels and clinical severity of COVID-19; Figure S4: Correlation between cytokine levels and pneumonia severity in COVID-19; Figure S5: The lack of association between cytokine levels and COVID-19 outcome; Figure S6: Correlation between tissue damage and autophagy markers in COVID-19; Figure S7: The diagnostic ability of p62 and cytokine levels in COVID-19; Figure S8: Correlation between p62 levels and COVID-19 comorbidities or severity/outcome; Figure S9: The effect of NSP5 and ORF3a on LC3-I levels in THP-1 cells; Figure S10: Original images of immunoblots from Figure 4a; Figure S11: Original images of immunoblots from Figure 5a,b; Figure S12: The effect of NSP5 and ORF3a on R848-induced cytokine expression; Figure S13: Original images of immunoblots from Figure 6b,c; Table S1: Clinical characteristics of COVID-19 patients; Table S2: Comparison of laboratory/clinical parameters of COVID-19 patients at admission and 7 days later.
