# Supplementary material for: Autophagy Receptor p62 Regulates SARS-CoV-2-Induced Inflammation in COVID-19
Source: Cells. 2023 Apr 28;12(9):1282. doi: 10.3390/cells12091282 (PMC10177105; doi:10.3390/cells12091282)
Supplement: Supplementary file 1 [file cells-12-01282-s001.zip › cells-2289691-supplementary.pdf]

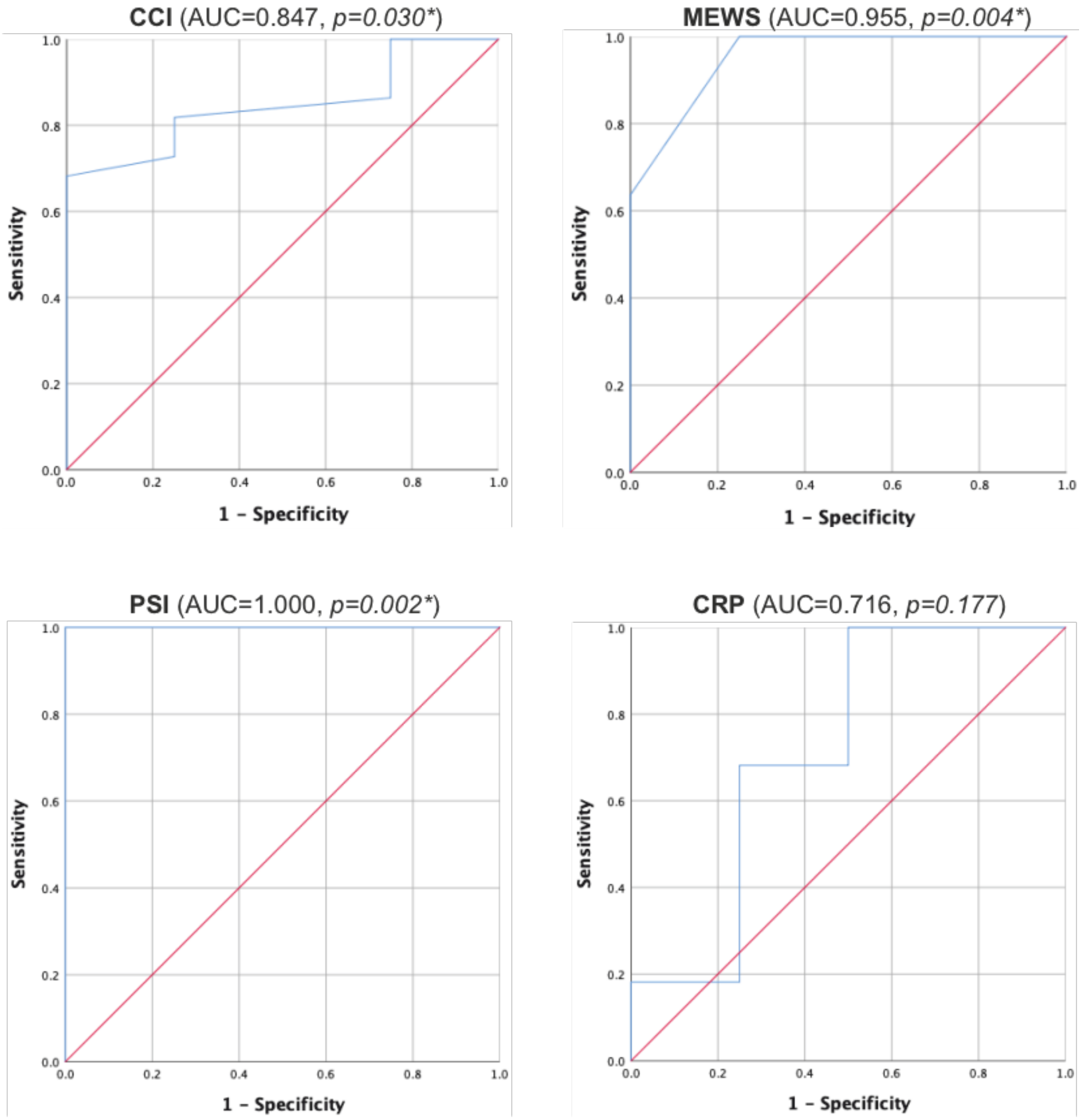

Figure S1. The ability of various parameters to predict COVID-19 outcome. Receiver operating characteristic (ROC) curve analysis of high Charlson Comorbidity Index (CCI), Modified Early Warning Score (MEWS), Pneumonia Severity Index (PSI), and blood C-reactive protein (CRP) values at hospital admission as predictors of poor COVID-19 outcome (*$p < 0.05$). AUC - area under the curve.

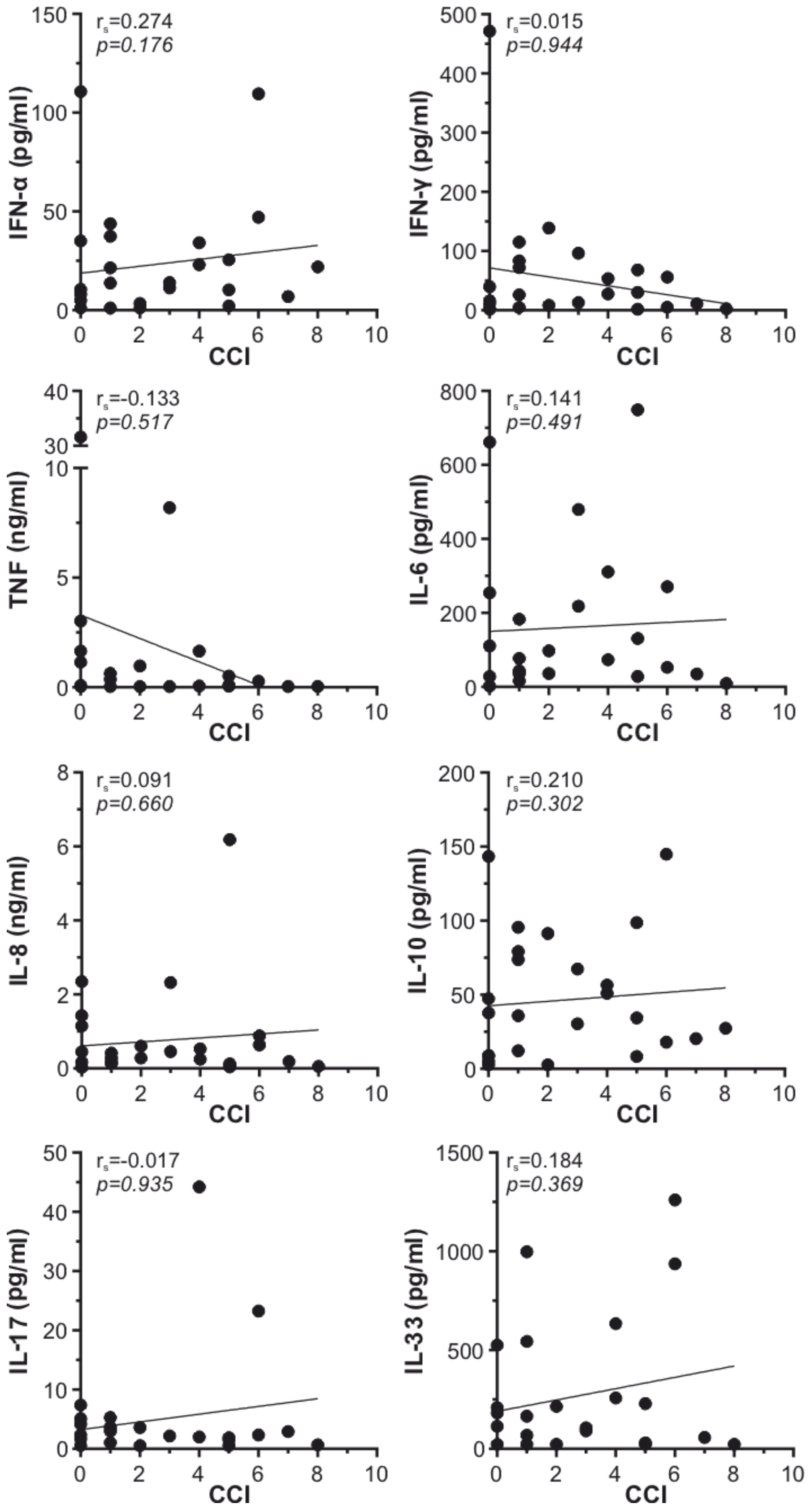

Figure S2. Correlation between cytokine levels and comorbidities in COVID-19. The correlation between Charlson Comorbidity Index (CCI) and blood plasma levels of cytokines in COVID-19 patients (n = 26) at hospital admission was assessed by the Spearman rank-order test (r_s - Spearman's correlation coefficient).

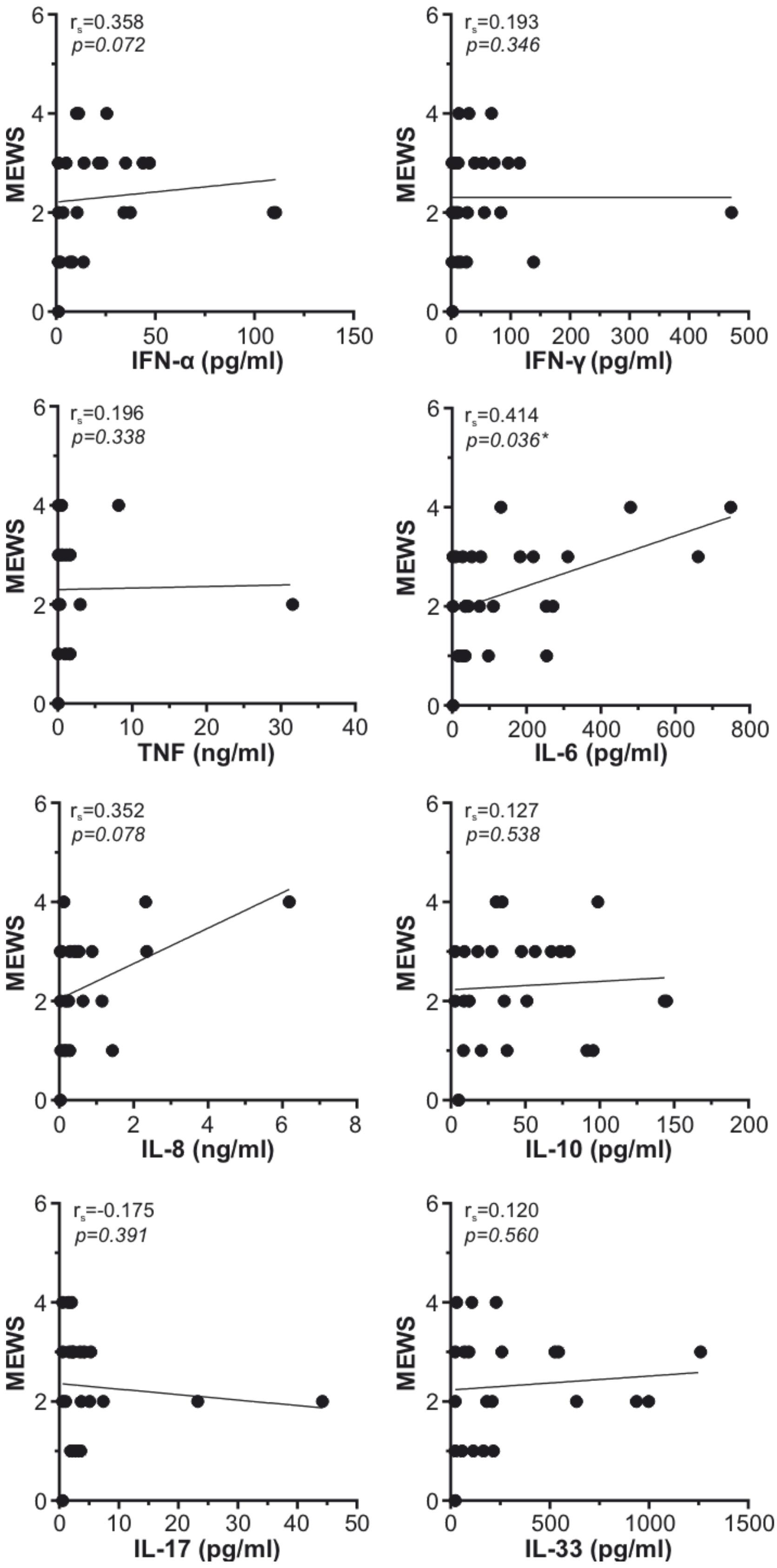

Figure S3. Correlation between cytokine levels and clinical severity of COVID-19. The correlation between Modified Early Warning Score (MEWS) as a measure of disease severity and blood plasma levels of cytokines in COVID-19 patients (n = 26) at hospital admission was assessed by Spearman rank-order test (r_s - Spearman's correlation coefficient, *$p < 0.05$).

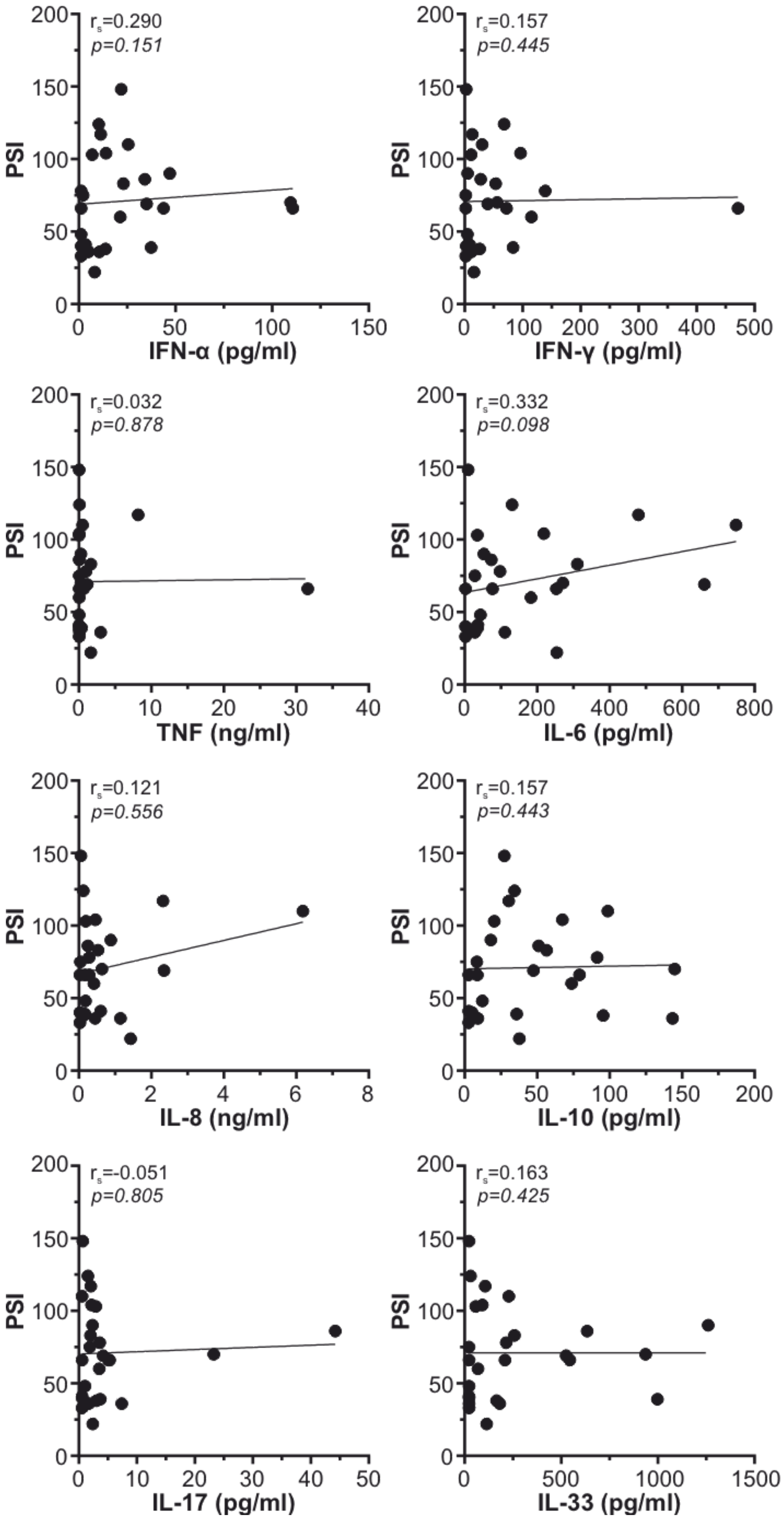

Figure S4. Correlation between cytokine levels and pneumonia severity in COVID-19. The correlation between the Pneumonia Severity Index (PSI) and blood plasma levels of cytokines in COVID-19 patients (n = 26) at hospital admission was assessed by the Spearman rank-order test (r_s - Spearman’s correlation coefficient).

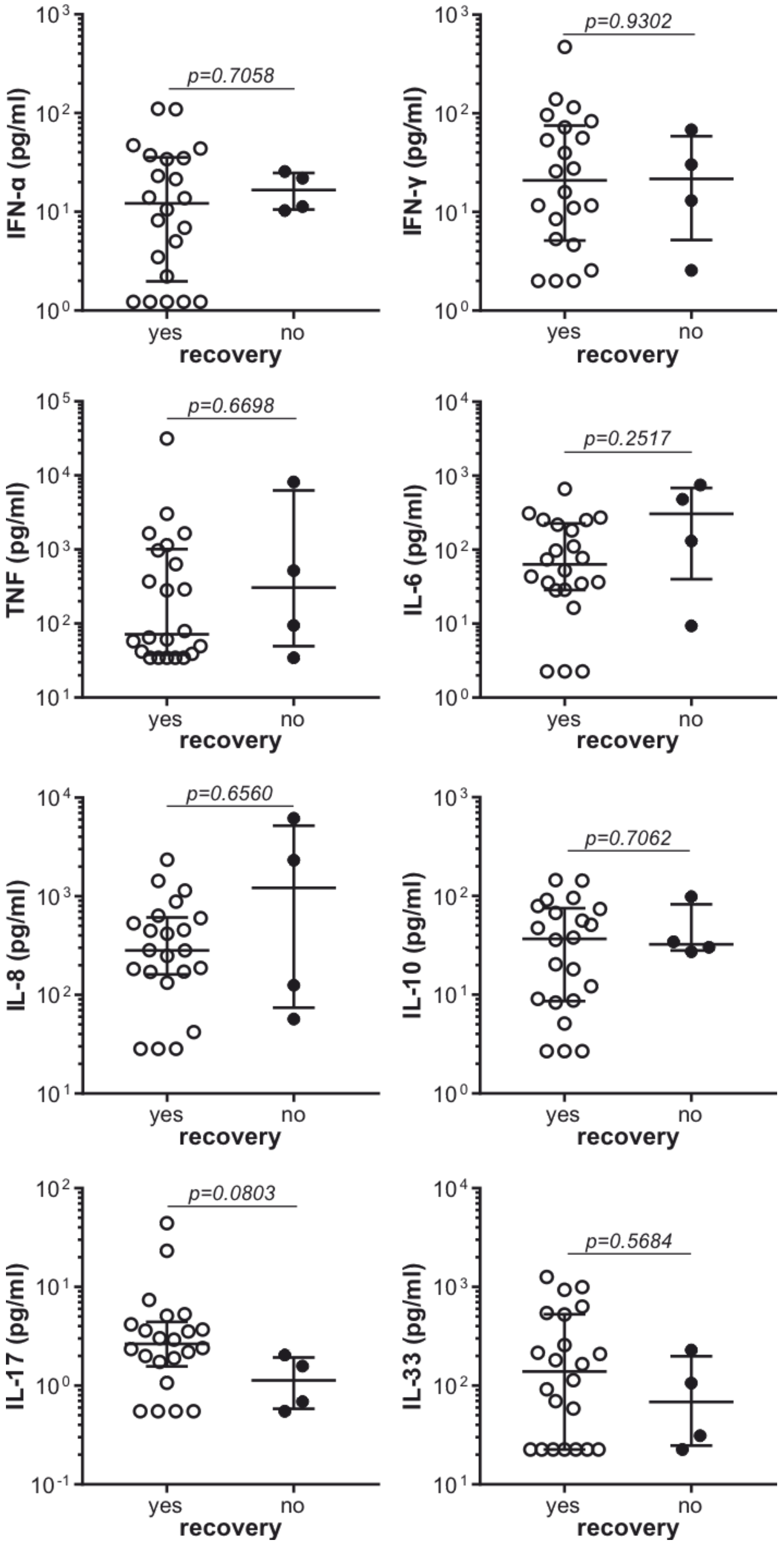

Figure S5. The lack of association between cytokine levels and COVID-19 outcome. Cytokine blood plasma levels at hospital admission were compared between COVID-19 patients who recovered (n = 22) and those who did not recover (n = 4) from the disease after 4 weeks (two-tailed Mann-Whitney U test; median and 25/75 percentile values are shown as lines).

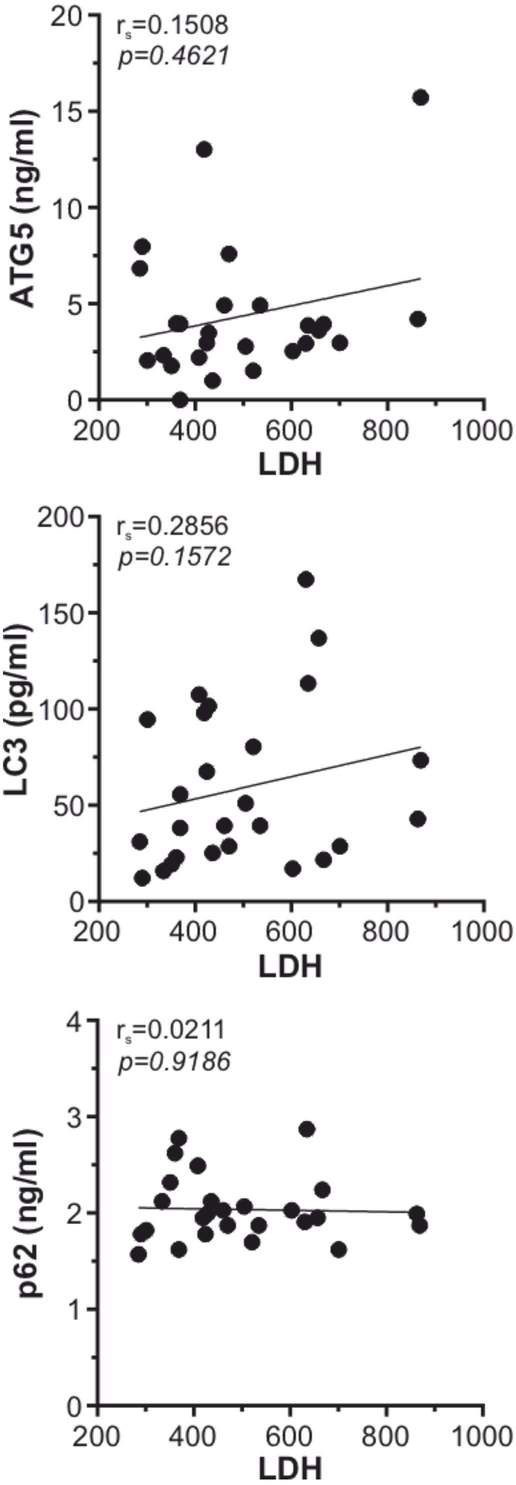

Figure S6. Correlation between tissue damage and autophagy markers in COVID-19. The correlation between blood plasma levels of cytokines and lactate dehydrogenase (LDH) as a measure of tissue damage in COVID-19 patients (n = 26) at hospital admission was assessed by Spearman rank-order test (r_s - Spearman's correlation coefficient).

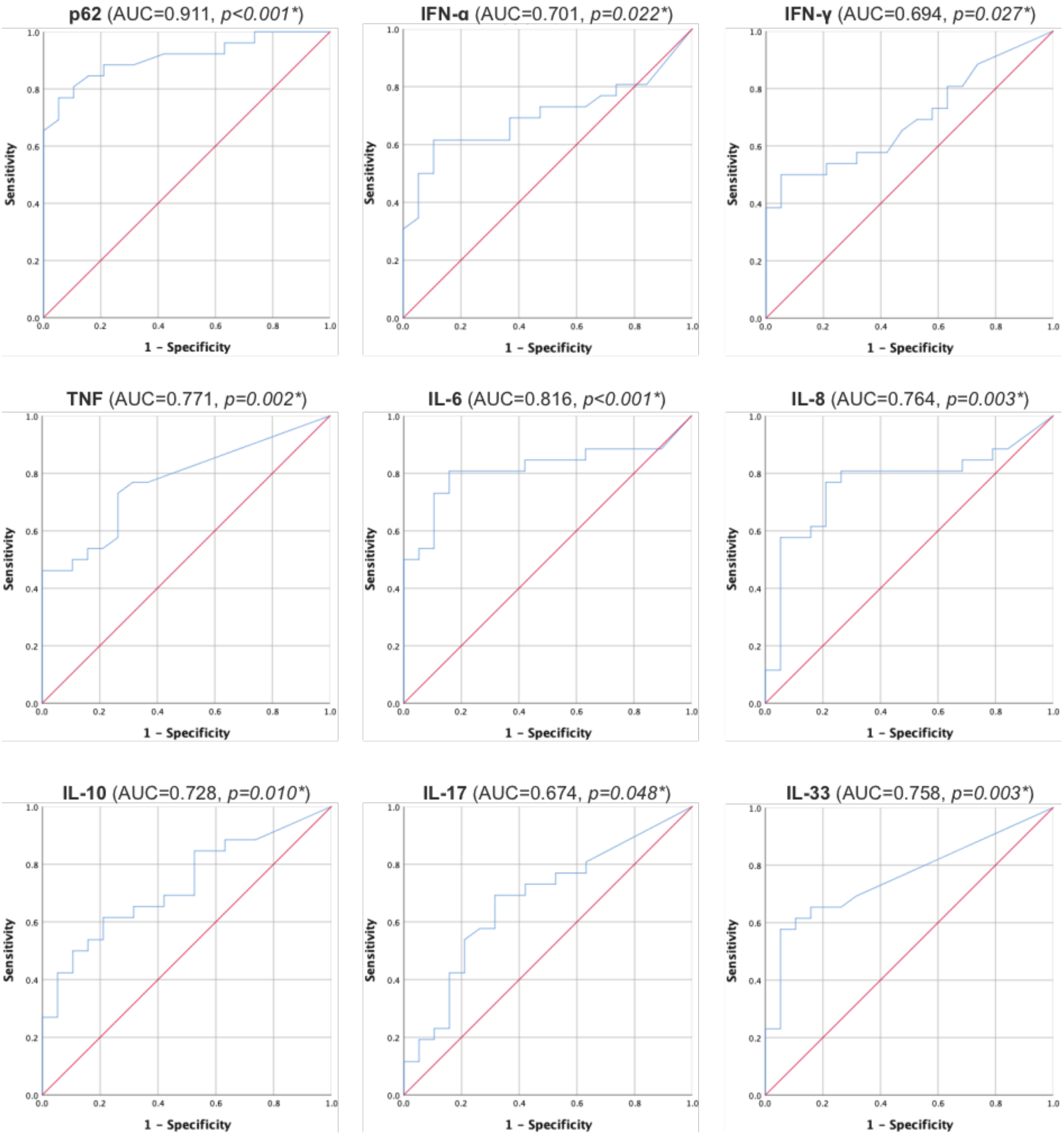

Figure S7. The diagnostic ability of p62 and cytokine levels in COVID-19. Receiver operating characteristic (ROC) curve analysis of the ability of low p62 and high cytokine values to discriminate COVID-19 patients (n = 26) from age/sex-matched healthy controls (n = 19). AUC - area under the curve.

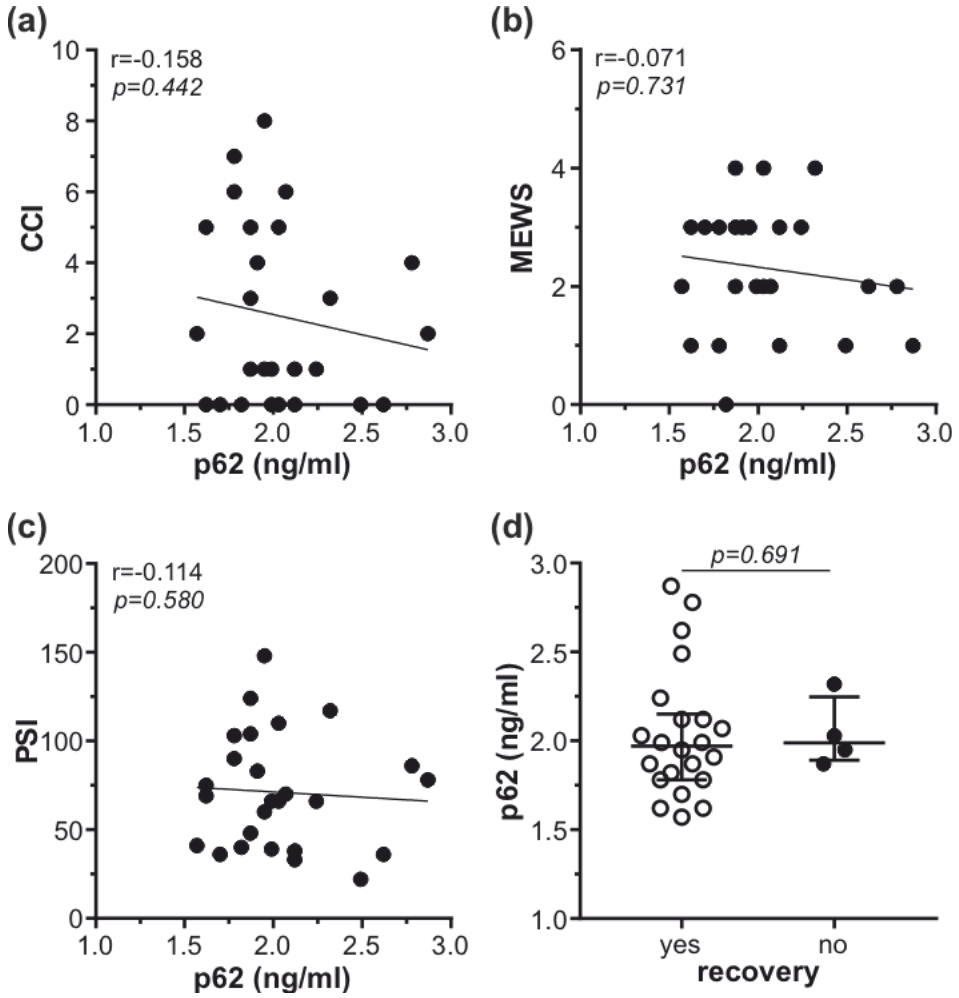

Figure S8. Correlation between p62 levels and COVID-19 comorbidities or severity/outcome. (a-c) The correlation between blood plasma levels of p62 and Charlson Comorbidity Index (CCI) (a), Modified Early Warning Score (MEWS) (b), or Pneumonia Severity Index (PSI) (c) in COVID-19 patients (n = 26) at hospital admission was assessed by Spearman rank-order test (r_s - Spearman's correlation coefficient). (d) Blood plasma levels of p62 at hospital admission were compared between COVID-19 patients who recovered (n = 22) and those who did not recover (n = 6) from the disease after 4 weeks (two-tailed Mann-Whitney U test; median and 25/75 percentile values are shown as lines).

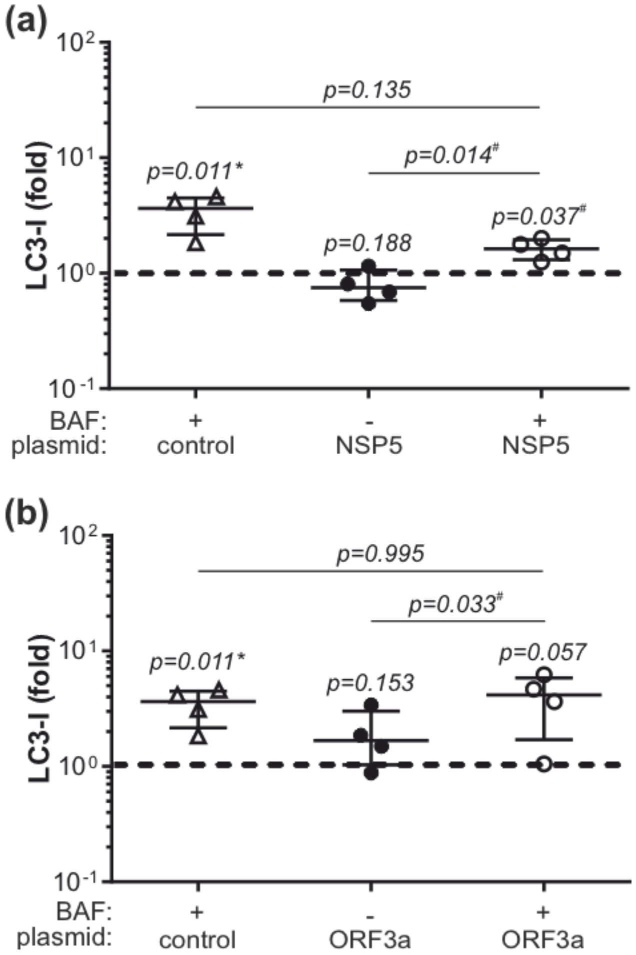

Figure S9. The effect of NSP5 and ORF3a on LC3-I levels in THP-1 cells. (a, b) THP-1 transfected with control plasmid or plasmids encoding NSP5 (a) or ORF3a (b) were incubated in a cell culture medium with 5% FBS, in the presence or absence of lysosomal inhibitor bafilomycin A1 (BAF; 50 nm) during the last 6 h of 48 h incubation period. Intracellular LC3-I was detected by immunoblotting (blots are shown in Figure 5a, b), and its levels were quantified by densitometry and expressed relative to β-actin as a loading control. The data from four independent experiments are presented as fold change relative to the control value (dashed line) obtained in cells transfected with control plasmid and not treated with BAF (median and 25/75 percentile values are shown as lines; *$p < 0.05$, two-tailed one-sample t-test vs. untreated control; $^{\#}p < 0.05$, two-tailed paired t-test).

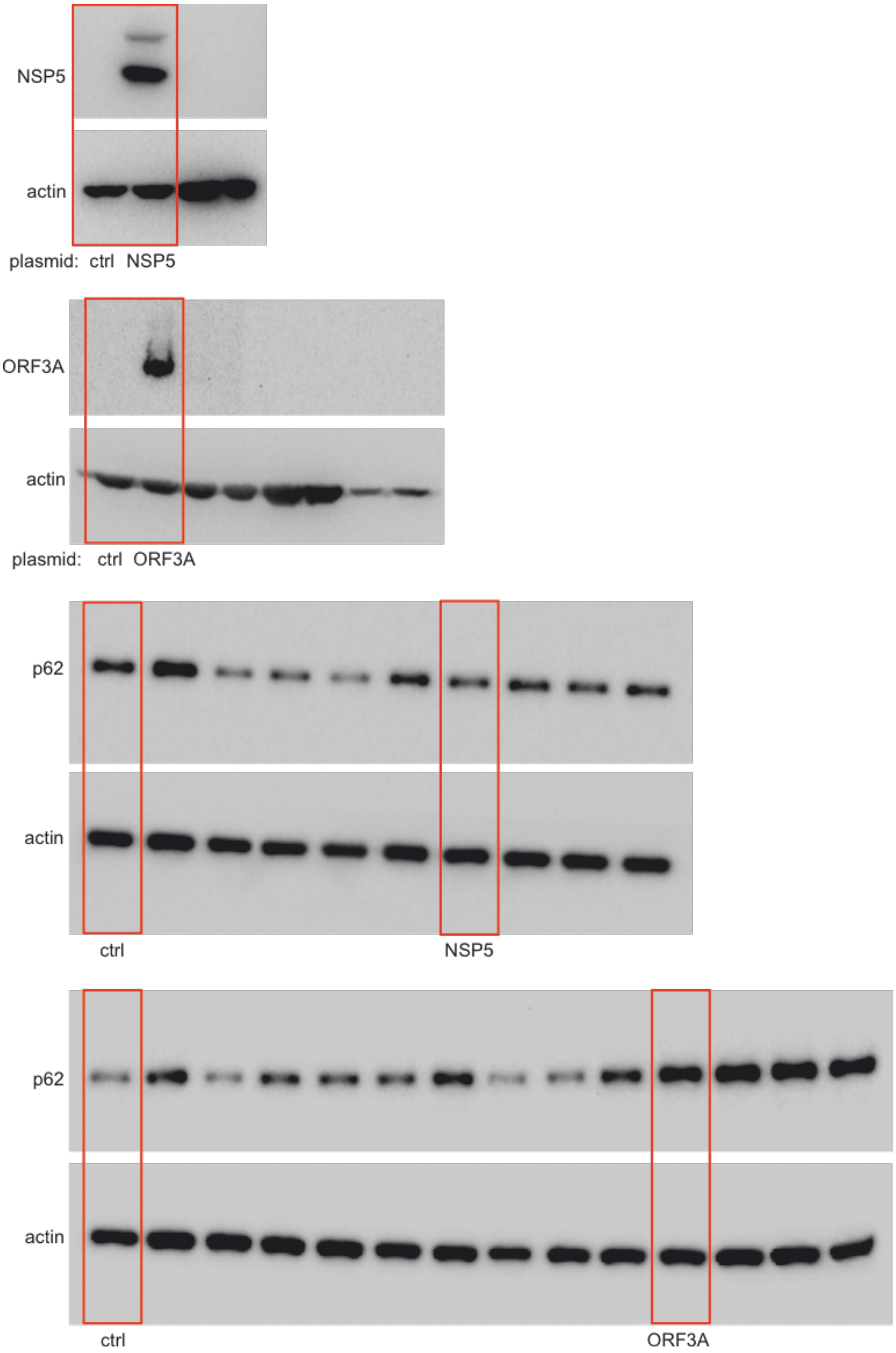

Figure S10. Original images of immunoblots from Figure 4a.

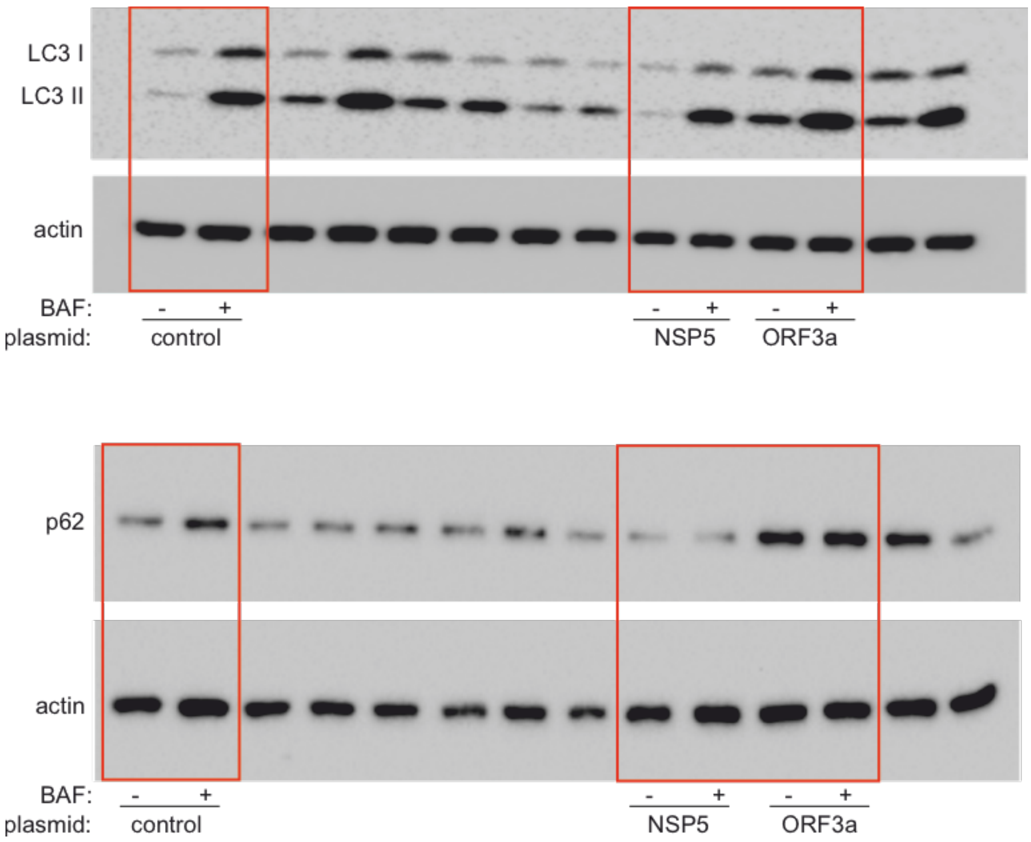

Figure S11. Original images of immunoblots from Figure 5a, b.

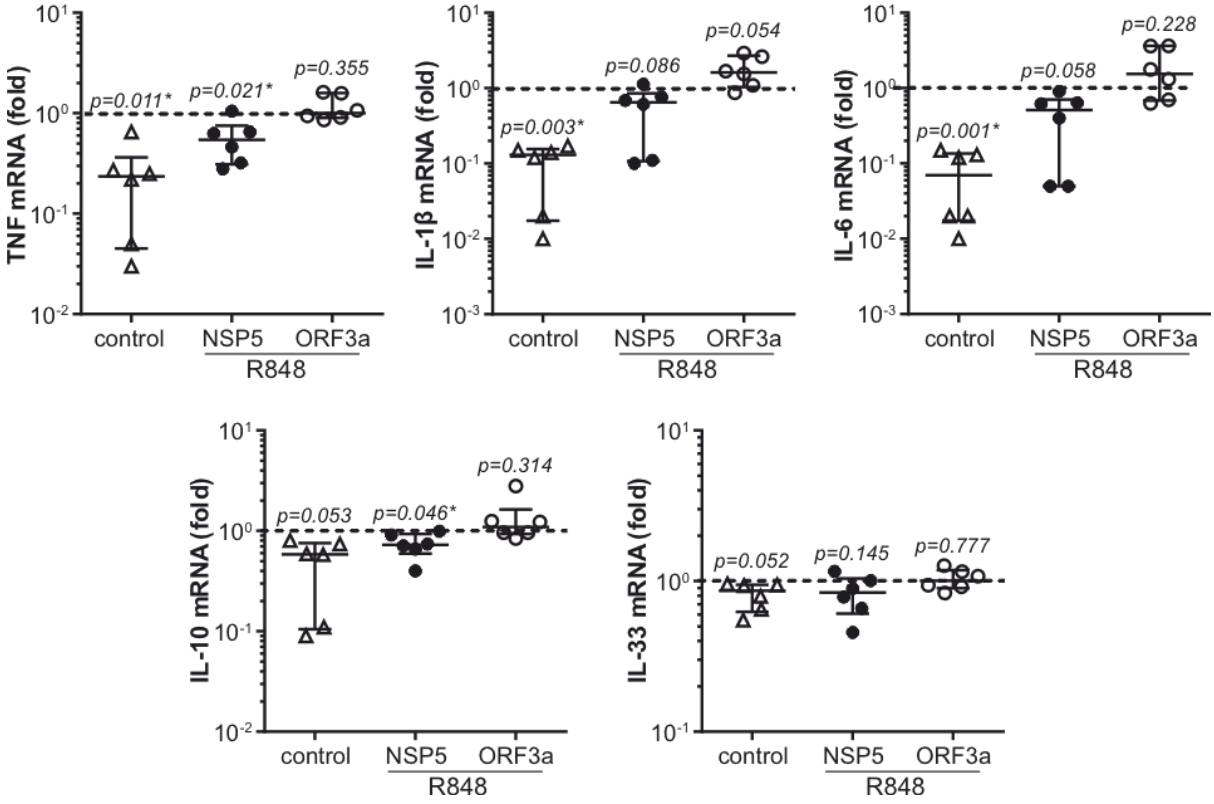

Figure S12. The effect of NSP5 and ORF3a on R848-induced cytokine expression. THP-1 cells were transfected with control plasmid or plasmids encoding NSP5/ORF3a and stimulated with TLR7/8-agonist resiquimod (R848; 10 µM). After 48 h, the cytokine (TNF, IL-1β, IL-6, IL-10, IL-33) mRNA levels were measured by RT-qPCR. The data from six independent experiments are presented as fold change relative to cytokine concentration in control plasmid-transfected cells treated with R848 (dashed line) (median and 25/75 percentile values are shown as lines; $^*p < 0.05$, two-tailed one-sample t-test).

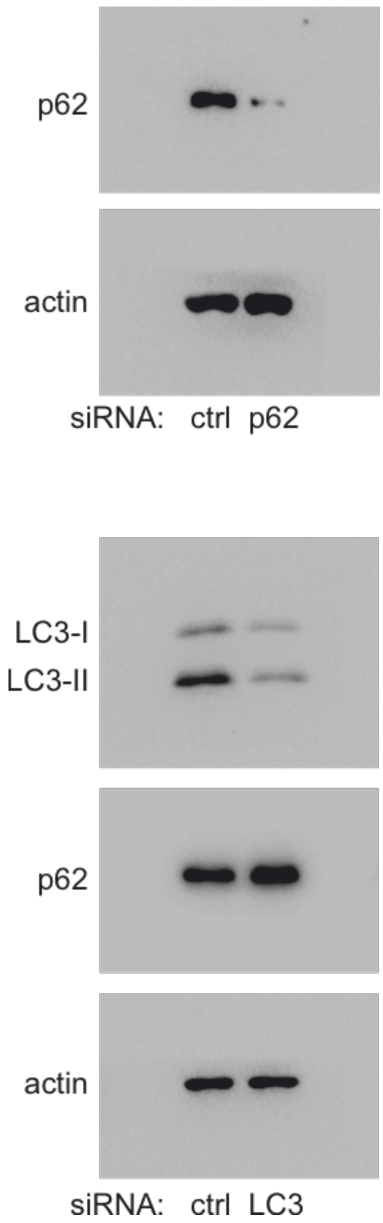

Figure S13. Original images of immunoblots from Fig. 6b, c.

Table S1. Clinical characteristics of COVID-19 patients.

Clinical characteristics	No. of patients
Comorbidities	17 (65.4%)
cardiovascular diseases	10 (38.5%)
diabetes	6 (23.1%)
hyperlipemia	4 (15.4%)
malignancy	2 (7.7%)
asthma	2 (7.7%)
COPD	3 (11.6%)
Symptoms	26 (100%)
fever	24 (92.3%)
cough	10 (38.5%)
fatigue	4 (15.4%)
diarrhea	17 (65.4%)
vomiting	9 (34.6%)
chest pain	4 (15.4%)
dyspnea	9 (34.6%)
myalgia	8 (30.8%)
headache	4 (15.4%)
Pneumonia	24 (92.3%)
unilateral	8 (30.8%)
bilateral	16 (61.5%)
Disease severity	
moderate	12 (46.1%)
severe	8 (30.8%)
critical	6 (23.1%)
Antibiotics before diagnosis	16 (61.5%)
Therapy after diagnosis	26 (100%)
antibiotics	26 (100%)
fraxiparine	26 (100%)
hydroxychloroquine	23 (88.5%)
corticosteroids	11 (42.3%)
tocilizumab	4 (15.4%)
favipiravir	5 (19.2%)
oxygen	14 (53.8%)
Disease outcome	
recovered, discharged home	22 (84.6%)
transferred to ICU	3 (11.6%)
deceased	1 (3.8%)

COPD, Chronic Obstructive Pulmonary Disease; ICU, Intensive Care Unit

Table S2. Comparison of laboratory/clinical parameters of COVID-19 patients at admission and 7 days later.

Laboratory/clinical parameters	day 0	day 7	p value
Leukocytes ($\times 10^9$/L)	5.3 (4.1-6.2)[a]	6.8 (6.1-8.1)	0.007*[b]
Neutrophils ($\times 10^9$/L)	3.0 (2.5-4.3)	3.4 (2.9-6.3)	0.335
Lymphocytes ($\times 10^9$/L)	1.2 (0.8-1.8)	1.9 (1.2-2.2)	0.002*
Monocytes ($\times 10^9$/L)	0.4 (0.3-0.6)	0.6 (0.4-0.8)	0.070
Thrombocytes ($\times 10^9$/L)	185 (177-314)	285 (230-363)	0.003*
Erythrocytes ($\times 10^{12}$/L)	4.2 (3.9-4.6)	4.4 (4.0-4.7)	0.295
Hemoglobin (g/L)	120 (113-136)	124 (115-137)	0.173
CRP (mg/L)	35.9 (6.7-56.4)	3.3 (1.4-7.3)	<0.001*
LDH (IU/L)	449 (367-631)	390 (355-545)	0.107
MEWS	2 (1.75-3)	0 (0-0.25)	<0.001*
PSI	68 (40-93)	56 (41-85)	0.040*

[a]Median (interquartile range); [b]two-tailed Wilcoxon signed rank test; CRP, C-reactive protein; LDH, lactate dehydrogenase; MEWS, Modified Early Warning Score; PSI, Pneumonia Severity Index
